# Pre-treatment with compound Danshen dripping pills prevents lipid infusion-induced microvascular dysfunction in mice

**DOI:** 10.1080/13880209.2020.1790619

**Published:** 2020-07-20

**Authors:** Yanda Zhang, Jian Zhao, Ru Ding, Wenhao Niu, Zhiqing He, Chun Liang

**Affiliations:** Department of Cardiology, Changzheng Hospital, Second Military Medical University, Shanghai, China

**Keywords:** Traditional Chinese medicine, coronary flow reserve, leukocyte adhesion, FOXO1

## Abstract

**Context:**

Recent studies have shown compound Danshen dripping pills (CDDP) could improve microcirculation in ischemic/reperfusion injury and other microvascular disorders. The mechanism for CDDP’s role in microcirculation is not clear.

**Objective:**

To explore the protective effects of CDDP on microvascular dysfunction.

**Materials and methods:**

C57BL/6 male mice (6–8 weeks) were randomized into control, model and CDDP groups (*n* = 10), which were treated with normal saline or CDDP (105.30 mg/kg), respectively. Then, lipid emulsion and heparin were infused via mice jugular vein to establish systemic microvascular dysfunction model. Coronary flow reserve (CFR) and leukocytes adhesion on microvascular wall were measured. Relative CD11b and CD62L expression levels on neutrophils were measured by flow cytometric analysis. Expression level of forkhead box transcription factor O1 (FOXO1) mRNA was identified by real-time PCR.

**Results:**

Lipid infusion significantly attenuated the CFR (1.84 ± 0.14 vs. 2.65 ± 0.02) and increased the number of leukocytes adherent to microvascular wall in cremaster (4067.00 ± 581.20 cells/mm^2^ vs. 10.67 ± 4.81 cells/mm^2^). The expression level of CD11b and FOXO1 in neutrophils was also up-regulated by lipid infusion. Pre-treatment with CDDP significantly improved CFR (2.57 ± 0.29 vs. 1.84 ± 0.14), decreased the number of leukocytes adherent to microvascular wall (2500.00 ± 288.70 cells/mm^2^ vs. 4067.00 ± 581.20 cells/mm^2^) and down-regulated CD11b and FOXO1 expression.

**Discussion and conclusions:** Pre-treatment with CDDP could prevent lipid infusion-induced systemic microvascular disorder including coronary and peripheral microvascular dysfunction. Down-regulated FOXO1 and decreased leukocyte adhesion might play an important role in the mechanisms of CDDP’s efficacy.

## Introduction

Microvascular dysfunction has been identified as an important cause of ischaemia in cardiovascular disease. Coronary microvascular dysfunction (CMD) is quite common in non-obstructive coronary artery disease and affects patients’ life quality (Safdar et al. 2020). However, there is still no effective therapy for CMD.

Compound Danshen dripping pills (CDDP), a patent traditional Chinese medicine, is widely used for relieving myocardial ischaemia in China and other countries such as South Korea, Mongolia, Singapore, Vietnam and Canada. CDDP includes three medicinal herbs, *Salvia miltiorrhiza* Bunge (Lamiaceae), *Panax notoginseng* (Burkill) F.H.Chen (Araliaceae) and borneol (Lv et al. 2019). Recent studies have shown that the active components of CDDP could improve microcirculation in ischemic/reperfusion injury and other microvascular disorders (Wang et al. 2009; Yang et al. 2015). However, the mechanism for CDDP’s role in microcirculation is still not fully clear.

Previous studies demonstrated that increased serum concentrations of free fatty acids could impair systemic microvascular function including coronary microcirculation (de Jongh et al. 2004; Yasu et al. 2018). In addition, microvascular dysfunction model can be successfully created in mice by lipid emulsion and heparin infusion to acutely elevate serum free fatty acid levels (Turpin et al. 2009).

In our study, we established a mouse model of microvascular dysfunction by lipid infusion to investigate CDDP’s effects on coronary and peripheral microcirculation.

## Materials and methods

### Animals and reagents

C57BL/6 mice were obtained from Medical Animal Center, Second Military Medical University. CDDP was purchased from Tianshili Pharmaceutical Group Co. Ltd. (Tianjin, China). Lipid emulsion was purchased from Huarui Pharmaceutical Co. Ltd. (Wuxi, China). Rat anti-mouse CD11b and CD62L were purchased from BD Bioscience (San Diego, CA). Primers were purchased from Ruijie Biotechnology Company (Shanghai, China).

The entire experimental protocol used in the study was carefully checked and approved by the Animal Experiment Ethics Committee of Second Military Medical University. All operations were performed under anaesthesia, and all possible efforts were made to minimize suffering, in accordance with the ARRIVE guidelines on animal research.

### Establishment of microvascular dysfunction model

Thirty male C57BL/6 mice (20 ± 2 g) were randomized into control group (*n* = 10), model group (*n* = 10) and CDDP group (*n* = 10). Normal saline (NS) was given to mice in control group and model group via oral gavage for three days, while mice in CDDP group were treated with CDDP (105.30 mg/kg). Then, mice were anaesthetized with pentobarbital (40 mg/kg) and the jugular vein was catheterized with microcatheter. Afterwards, mice underwent a 6 h NS (control group) or lipid emulsion plus heparin infusion through micro-pump (model and CDDP groups) (Lipid, 20% Intralipid^®^, heparin 20 U/mL, 0.1 mL/h) (Tripathy et al. 2003).

### Coronary flow reserve (CFR) measurement

After lipid infusion, mice were anaesthetized by isoflurane immediately. The hair of mice was shaved or removed by Nair on the chest area just below the base of the left upper limb to measure left main coronary flow velocity. The coronary flow velocity signal was measured with a 20 MHz probe of Mouse Doppler (Indus Instruments, Webster, TX) on the chest area and focussed through the 2nd or 3rd intercostal space on the left side of the chest. Once the coronary signal was found, the micropositioner was used to optimize the location of the sample volume in the left main coronary artery. The hyperaemic flow can be achieved by increasing the level of isoflurane to 2.5% and baseline flow can be obtained after reducing isoflurane level to 1% (Chang et al. 2015). Data were measured by Doppler signal processing workstation. CFR was calculated as, CFR = P/R=*V*_high_/*V*_low_.

### Cremaster microcirculation assessment

Mice were anaesthetized with pentobarbital (40 mg/kg) just after lipid infusion. Intravital microscopy (BX51, Olympus, Tokyo, Japan) was used to record the cremaster venular blood flow and leukocytes adhesion in venules. Then, microvascular blood flow images and videos were recorded by OLYMPUS DP71 digital microscope camera. Venules 200 μm in length and ranging from 80 to 120 μm in diameter were chosen for analysing (Li et al. 2012; Wang et al. 2018). Quantification of microvascular blood flow velocity in cremaster and leukocyte adhesion in venules were completed by Image-Pro Plus 6.0 software (Media Cybernetic, Rockville, MD) (Zhang et al. 2019). Leukocyte flux and leukocyte endothelium interactions (rolling and adhesion) analysis was conducted as described by Wang et al. (2019). Briefly, rolling leukocyte flux was measured at indicated time points by counting the number of rolling leukocytes per 20 s passing a reference point in the microvessel and expressed as cells/min. Leukocyte rolling velocity was calculated by the velocity of 10 leukocytes rolling along endothelial cell lining (µm/s). Leukocyte adhesion (stationary for 20 s) was counted in 100 µm long vascular segments and expressed as number of adherent cells per square millimetre. Diameters were measured in micrometre perpendicularly to the vessel path. Venular wall shear rate was calculated based on the Newtonian definition: Wall shear rate = 8 (red blood cell velocity/venular diameter) (House and Lipowsky 1987).

### Flow cytometric analysis

Blood was drawn from the great saphenous vein of the mice after lipid infusion. Ammonium chloride potassium (ACK) lysis buffer was used for erythrocyte removal. Blood sample was centrifuged for 10 min at 3000 rpm and the plasma was then removed. Leukocytes were resuspended with 20 μL NS and were then incubated with the appropriate antibodies as previously described (McDonald et al. 2015). Rat anti-mouse CD11b and CD62L were diluted 1:5. Flow cytometry was performed on Cytek's upgraded DXP8 Calibur by staining with appropriate antibodies, and the data were analysed using FlowJo 10.0 software (Tree Star, Inc., Ashland, OR).

### Heart histological examination and ultrastructural analysis

Mice were euthanized when the lipid infusion was completed. Fresh heart sections were fixed in 4% paraformaldehyde, embedded in paraffin and stained with haematoxylin–eosin (HE). Pathologists examined sections to observe the changes of cardiomyocytes and microvessels.

Heart samples were fixed in 2.5% glutaraldehyde in 0.1 mol/L cacodylate buffer, then fixed in 1% osmium tetroxide and then embedded in Epon (Embed-812, Hatfield, PA). Ultrathin sections (80–100 nm) were obtained from selected areas using an ultrathin microtome (Leica UC7, Wetzlar, Germany), contrasted with uranyl acetate and lead citrate, and examined with a transmission electron microscope (TEM) (Tecnai G2 20, ST, FEI, Hillsboro, OR).

### Real-time PCR analysis

All mice were euthanized at the end of the experiment. Whole blood was collected for gene expression testing. Blood sample was centrifuged for 10 min at 3000 rpm and the plasma was then removed. Total RNA was extracted using RNAiso plus reagent (Takara, Kusatsu, Japan), and the purity of the RNA was assessed using A260/A280 ratio spectrophotometry. PrimeScript^®^ RT Master Mix kits were used for reverse transcription to obtain cDNA. A 10 μL reaction system was then prepared using the SYBR^®^ PremixEx Taq™ kit to measure the relative expression level of forkhead box transcription factor O1 (FOXO1) mRNA. Data were normalized to β-actin. All primers were obtained from Primer Bank and are listed in Table 1.

**Table 1.** The sequence of primer used for reverse transcription.

Gene	Forward	Reverse
β-actin	5′-TTCCTTCTTGGGTATGGAAT-3′	5′-GAGCAATGATCTTGATCTTC-3′
FOXO1	5′-TCGTACGCCGACCTCATCA-3′	5′-CTGTCGCCCTTATCCTTGAAGT-3

### Statistical analysis

Data were presented as mean ± standard deviation. GraphPad Prism 5.01 (La Jolla, CA) and SPSS 25.0 (IBM, Inc., Armonk, NY) were deployed to compare the difference among groups by one-way analysis of variance (ANOVA). Two-side *p* < 0.05 was considered statistically significant.

## Results

### Lipid infusion induced microvascular dysfunction

**Figure 1.**
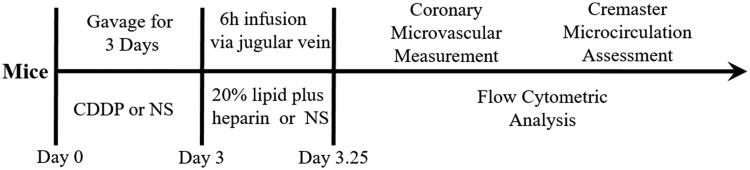
The experimental scheme.

**Figure 2.**
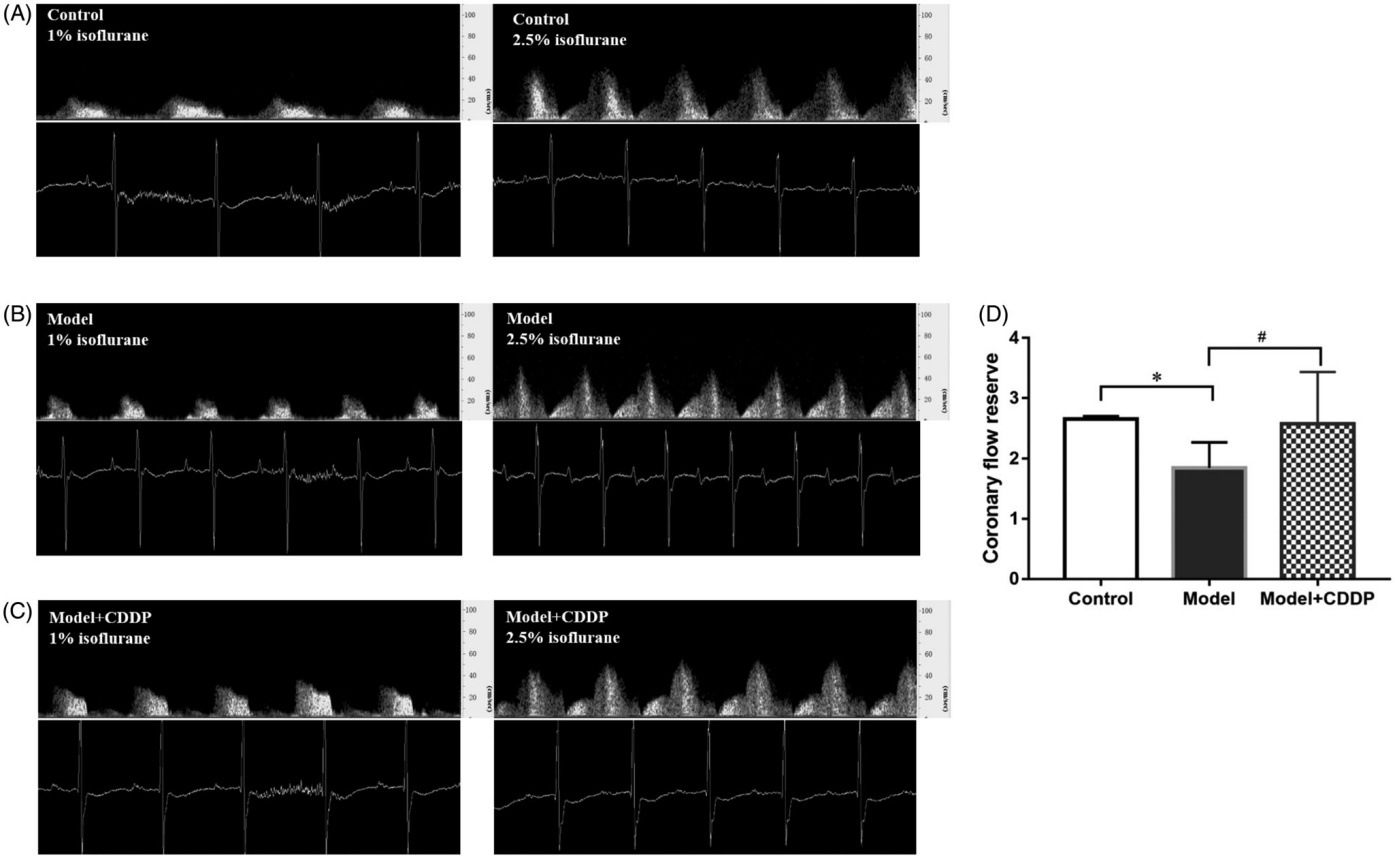
Pre-treatment with CDDP improved coronary flow reserve. Data are mean ± SD from 10 mice. *p < 0.05, model vs. control group. ^#^*p* < 0.05, model vs. model + CDDP group.

**Figure 3.**
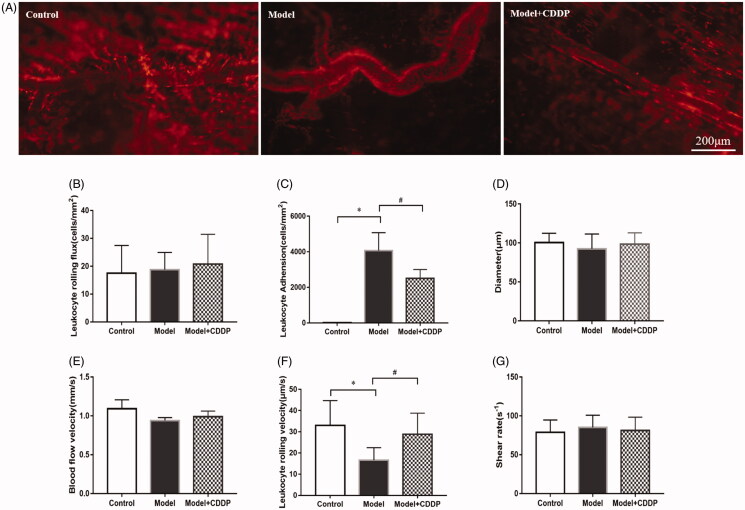
Pre-treatment with CDDP reduced leukocytes adhesion. Data are mean ± SD from 10 mice. *p < 0.05, model vs. control group. ^#^*p* < 0.05, model vs. model + CDDP group.

**Figure 4.**
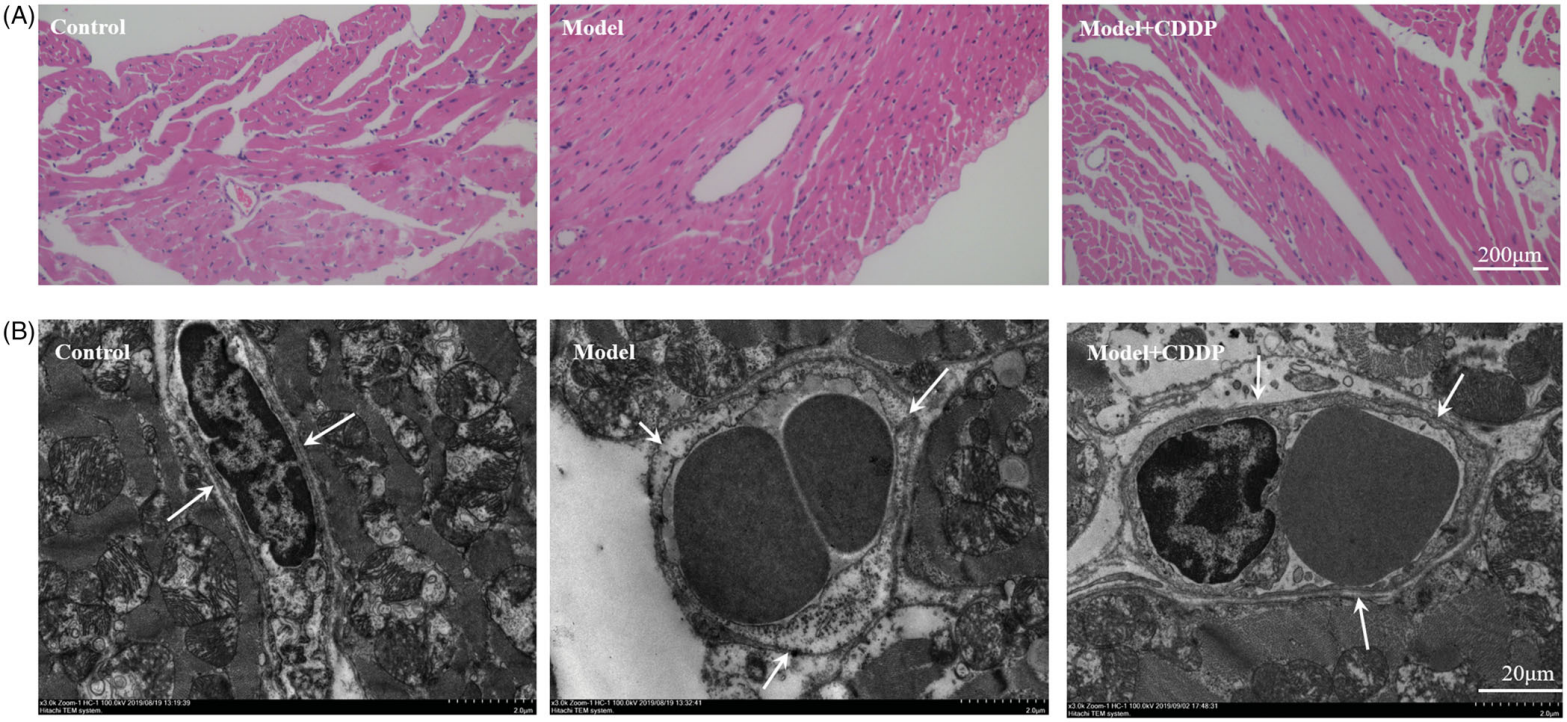
Pre-treatment with CDDP alleviated endothelial injury. (A) ×200 light microscope and (B) transmission electron microscope showed CDDP alleviated the oedema of endothelial cells (white arrow).

**Figure 5.**
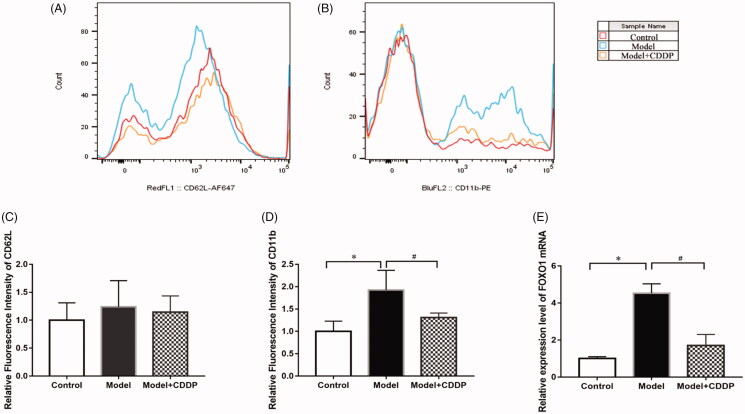
CDDP improved microcirculation via down-regulating CD11b and FOXO1 expression. (A, C) CD62L; (B, D) CD11b; (E) FOXO1.

The experimental scheme is shown clearly in Figure 1. In comparison with mice in control group, lipid infusion significantly attenuated the CFR (1.84 ± 0.14 vs. 2.65 ± 0.02) (Figure 2) and increased the number of leukocytes adhesion to microvascular wall in cremaster (4067.00 ± 581.20 cells/mm^2^ vs. 10.67 ± 4.81cells/mm^2^) (Figure 3). Lipid infusion also significantly decreased the leukocyte rolling velocity. But it had no effect on the blood flow velocity, shear rate, leukocyte rolling flux and diameter. There was no significant change in HE sections under ×200 microscope (Figure 4(A)). Lipid infusion caused obvious oedema of endothelial cells in model group (Figure 4(B)). Lipid infusion had no effect on the expression level of CD62L (Figure 5(A,C)), but the expression level of CD11b on neutrophils was up-regulated by lipid infusion (Figure 5(B,D)).

### CDDP improved CFR and reduced leukocytes adhesion

Pre-treatment with CDDP significantly improved the CFR (2.57 ± 0.29 vs. 1.84 ± 0.14) (Figure 2). Besides, CDDP decreased the number of leukocytes adherent to microvascular wall (2500.00 ± 288.70 cells/mm^2^ vs. 4067.00 ± 581.20 cells/mm^2^) (Figure 3) and down-regulated CD11b expression level (Figure 5(B,D)).

### CDDP alleviated endothelial injury

TEM showed endothelial damage in model mice and nearly intact endothelia in CDDP group (Figure 4(B)). CDDP alleviated the oedema of endothelial cells (Figure 4(B)).

### CDDP down-regulated FOXO1 mRNA expression

Real-time PCR showed that the relative expression level of FOXO1 in leukocytes of model mice was about 4.5-fold compared with mice in control group. Pre-treatment with CDDP restored the expression of FOXO1 (Figure 5(E)).

## Discussion

CMD has been identified as an important cause of myocardial ischaemia, which is common in patients with stable angina. It was shown that 68% of non-obstructive coronary artery disease patients had an abnormality in more than one parameter of CMD (Corcoran et al. 2018). CMD is associated with increased risk of adverse cardiovascular events like myocardial infarction and this has aroused more and more attention in cardiovascular field (Taqueti 2020). However, there is still a need for effective CMD therapy (Schindler and Dilsizian 2020).

CDDP is a modernized pharmacological preparation based on traditional Chinese medicine therapeutic theory. It came into market in 1994 and has completed its II phase and III phase clinical trials in the USA (Liao et al. 2019). The active pharmacodynamic substances consist mainly of phenolic acids, saponins and borneol. As previously described, CDDP has inhibiting cardiac cell apoptosis, anti-oxidative, anti-inflammatory and endothelium-protective effects (Jun et al. 2014; Chinese Geriatrics Society 2017). CDDP could enhance a metabolic shift towards fatty acids metabolism in order to modulate the perturbed energy metabolism in a rat model of acute myocardial ischaemia, and it could also protect cardiomyocytes and inhibit apoptosis by activating Akt-eNOS signalling pathway (Ren-an et al. 2014; Guo et al. 2016). Clinical trial has presented that CDDP as a supplement to general therapy for microvascular angina can increase treatment efficacy and plasma NO level (Fang 2015).

In the present study, systemic microvascular dysfunction model was successfully established by lipid infusion, including CMD and peripheral microvascular dysfunction. Pre-treatment with CDDP significantly improved coronary microvascular disorder caused by lipid infusion. CFR value was reserved in CDDP group while CFR was heavily reduced in the other two groups. CFR is a physiological index for the assessment of myocardial flow impairment due to focal or microcirculatory coronary artery disease (Stegehuis et al. 2020). TEM also revealed that pre-treatment with CDDP alleviated the oedema of microvascular endothelial cells. These results proved that CDDP is a promising medicine for CMD.

As to the mechanisms of CDDP in improving microvascular dysfunction, down-regulated FOXO1 and decreased leukocyte adhesion might play important roles. We observed upregulated FOXO1 and leukocytes activation in model group. Pre-treatment with CDDP restored this and ameliorated the outcome of mice in the CDDP group. CDDP downregulated the expression of FOXO1 and reduced the leukocyte adhesion molecule CD11b. Thus, leukocyte adhesion and inflammation in microcirculation were controlled and microvascular function was improved. In fact, inflammation is a well-known aetiology of microvascular dysfunction (Camici et al. 2015). Over expression of FOXO1 upregulates TLR2/4, which would increase the expression of CD11b and enhance neutrophil mediated inflammation through increasing production of inflammatory cytokines (TNF, IL-1, etc.) (Dong et al. 2017). Previous study also found pre-treatment with CDDP 100 mg/kg before ischaemia-reperfusion stimulation could significantly improve the CMD in rats, including increased coronary blood flow, decreased albumin leakage and down-regulated expression level of CD11b on neutrophils (Zhao et al. 2010).

Our study successfully established a microvascular dysfunction model and proved the efficacy of CDDP for microvascular dysfunction, which suggested that CDDP could be an effective drug for CMD. However, our study still has certain limitations. First, we did not investigate the microvascular structure changes of other organs such as kidney and retina. Second, the manipulation of FOXO1 via pharmacological or genetic methods was not conducted to determine the precise role of FOXO1 in the protective action of CDDP on microvascular dysfunction. Third, only short-term effects of CDDP were identified. The long-term pharmaceutical effects of CDDP on microvascular dysfunction remain unclear. Further studies are needed to establish the long-term efficacy of CDDP on microvascular dysfunction.

## Conclusions

Pre-treatment with CDDP could prevent lipid infusion-induced systemic microvascular disorder including coronary and peripheral microvascular dysfunction. Down-regulated FOXO1 and decreased leukocyte adhesion might play an important role in the mechanisms of CDDP’s efficacy.
